# Polymorphisms of cytochrome P450 1A1, glutathione s-transferases M1 and T1 genes in Ouangolodougou (Northern Ivory Coast)

**DOI:** 10.1590/S1415-47572010005000059

**Published:** 2010-09-01

**Authors:** Alfredo Santovito, Claudio Burgarello, Piero Cervella, Massimiliano Delpero

**Affiliations:** 1 Department of Animal and Human Biology, University of Turin, TorinoItaly; 2 Biomedical Laboratory of the Catholic Mission Father P. Pianzola, OungolodougouIvory Coast

**Keywords:** *CYP1A1*, *GSTM1*, *GSTT1*, genetic polymorphism, Ouangolodougou

## Abstract

In this study, the frequencies of *CYP1A1*, *GSTM1*, and *GSTT1* gene polymorphisms were determined in 133 healthy individuals from Ouangolodougou, a small rural town situated in the north of the Ivory Coast. As appeared in several published studies, ethnic differences in these frequencies have been found to play an important role in the metabolism of a relevant number of human carcinogens. In the studied sample, the frequencies of Ile/Ile (wild type), Ile/Val (heterozygous variant), and Val/Val (homozygous variant) *CYP1A1* genotypes were 0.271, 0.692, and 0.037, respectively. Frequencies of *GSTM1* and *GSTT1* null genotypes were 0.361 and 0.331, respectively. No significant differences were noted between men and women. In contrast to published data for Africans, *CYP1A1* *Val Allele frequency (0.383) was significantly high (p < 0.001) in this specific population. For the *GSTT1* null genotype, no differences were found between the studied and other African populations, the contrary to what occurred for the *GSTM1* null genotype in relation to Gambia and Egypt.

Several polymorphic genetic systems have been used to study human genetic variation. Herein we report for a sample from the Ivory Coast on the frequencies of three polymorphic metabolic genes that have been found to play an important role in the metabolism of a relevant number of human carcinogens: the Cytochrome P450 1A1 (*CYP1A1*), the Glutathione S-transferases (*GST*) *T1* and the *GSTM1* genes. Several published studies pointed to ethnic differences in the frequencies of gene polymorphisms, shown to be associated with several types of cancer. The *CYP1A1* gene is a component of the phase I cytochrome P450 superfamily, which plays a primary role in the metabolism of polycyclic aromatic hydrocarbons. Its gene product catalyses the first step in the conversion of several environmental carcinogens into their ultimate DNA-binding carcinogenic form (for a review see Indulski and Lutz, 2000). The human *CYP1A1* gene is highly polymorphic. In certain populations, such as the Japanese and Chinese, the amino acid substitution CYP1A1 Ile(462)Val has been reported to be associated with a higher risk of certain types of cancer, as lung cancer (Chen *et al.*, 2001), although in other populations, evidence of this association lacks support (McGrath *et al.*, 2007). These contradictory results could be explained by the distinct genetic backgrounds of the investigated populations, as well as other non-genetic factors.

Glutathione S-transferases, found in virtually all eukaryotes, represent one of the major groups of phase II detoxifying enzymes, having evolved to provide organic protection against toxic substances present in food and the environment (Nebert *et al.*, 1996). The mechanism involves the binding of glutathione to insoluble electrophilic substrates, thereby converting them into a soluble form (Perera, 1997). *GSTT1* and *GSTM1* genes have long been known to be polymorphic in humans by the deletion of a segment of DNA, with the subsequent lack of protein synthesis in homozygous individuals. As a result of deletions at either of the two loci, and the consequent reduced detoxification of xenobiotics, an individual may become susceptible to diseases produced by toxic substances present in the environment (Roy *et al.*, 2001). Deletion polymorphism in *GST* genes (non-functional alleles) has been found to be associated with the development of certain types of cancer, such as oral cancer (Cha *et al.*, 2007), chronic myeloid leukaemia (Bajpai *et al.*, 2007), bladder cancer (Ouerhani *et al.*, 2006), and colon cancer (Gertig *et al.*, 1998). Although variability in the distribution of *GSTM1* and *GSTT1* null genotypes has been reported in diverse ethnic groups (Nelson *et al.*, 1995; Garte *et al.*, 2001), sparse data are available for West Africa (Wild *et al.*, 2000). The aim herein was to investigate the genetic variability at *CYP1A1* and *GSTs* loci in an African population from the Ivory Coast, for which no data has been published to date on metabolic gene polymorphisms.

Sampling was undertaken in Ouangolodougou, a small rural town of 20,000 inhabitants located in the north Ivory Coast, this representing the last frontier zone between the Ivory Coast, Mali and Burkina Faso. One hundred and thirty-three individuals (62 males and 71 females, mean age 31.9 ± 14,5) were analyzed. All were healthy volunteers and had received prior information regarding the study.

Peripheral blood samples (5-10 mL venipuncture) were collected in heparinized vacutainers and stored at -20 °C. The Chelex^®^ solution protocol was used to extract DNA, as described by Walsh *et al.* (1991). *GSTM*, *GSTT1 and CYP1A1* genotypes were defined by polymerase chain reaction (PCR), using primers and reaction profiles as described by Zhong *et al.* (1993), Pemble *et al.* (1994) and Chen *et al.* (2001), respectively. PCR reactions were carried out in a total volume of 25 μL containing 10 ng of DNA (template), with a final concentration of 1X Reaction Buffer, 1.5 mM of MgCl_2_, 5% of DMSO, 250 μM of dNTPs, 0.5 μM of each primer, and 1 U/sample of Taq DNA polymerase (Fischer, U.S.). PCR products were separated by electrophoresis on a 3% agarose gel and visualized by ethidium bromide staining. The expected size of amplified *CYP1A1* products was 162 base pairs (bp), whereas in the case of *GSTM1*, *GSTT1* and β*-globine* (used as internal control), the expected sizes were 230, 480 and 110 bp, respectively. Homozygous null genotypes of *GSTM1* and *GSTT1* were determined by identifying the negative band for each size (with the simultaneous presence of the positive control), whereas positive bands meant the sample was homo- or heterozygous for the indicated alleles. A genotype with homozygous deletion of the *GST* genes is denominated “*GST*-null”, whereas a genotype having at least one copy of the gene is “*GST*-positive”. In order to validate the obtained results, about 20% of the total sample (n = 30) was genotyped independently by a second researcher.

Allele and genotype frequencies of the *CYP1A1* gene were calculated using GENEPOP Version 4.0 (Raymond and Rousset, 1995). Hardy-Weinberg equilibrium (HWE) and comparisons among age-groups were evaluated by the Chi-square (χ^2^) test with a 95% confidence interval. Observed allele frequencies were also compared with those reported for other populations worldwide, through the analysis of contingency tables also by χ^2^-testing.

The frequencies of *CYP1A1* and *GSTs* allelic polymorphisms in a sample from the north Ivory Coast population, were analyzed. No significant differences were found between men and women in the allele frequency of each gene (p > 0.05), thus possibly implying the absence of differences by sex in these detoxifying enzymes.

The observed frequencies of the *CYP1A1* Ile/Ile (wild type), Ile/Val (heterozygous variant) and Val/Val (homozygous variant) genotypes were 0.271, 0.692, and 0.037, respectively (Table 1). Differences in *CYP1A1* allele frequency distribution were reported among various ethnic groups (Garte, 1998; Garte *et al.*, 2001). In the studied Ivory Coast population, *Val allele frequency was significantly (p < 0.001) high (0.383), as compared to the few data published for Africans (Table 2). This difference in *CYP1A1* polymorphism distribution among Africans could probably be attributed to the different evolutionary histories of the studied ethnic groups, although, as this polymorphism is not neutral, the distinctive selective pressure exerted by chemicals on different populations cannot be excluded. The high heterozygosity frequency value found for *CYP1A1* gene (0.692) is congruent with many global surveys of genetic markers, these indicating that diversity in African populations is consistently greater than in other populations (Garrigan and Hammer, 2006). On the other hand, the excess of heterozygotes gave rise to a deviation from HWE (p < 0.05). Moreover, a selective pressure exerted by xenobiotics on this population cannot be excluded. Although this possibility is consistent with several studies showing the association of this polymorphic gene with risk factors for diseases (Chen *et al.*, 2001), convincing evidence to suggest a role of these polymorphisms in modulating susceptibility to cancer diseases, is lacking (McGrath *et al.*, 2007).

Differences in *GSTT1* and *GSTM1* frequencies in populations worldwide have already been described (Nelson *et al.*, 1995; Garte *et al.*, 2001). These differences show that *GST* genotype frequencies are distributed populationwise according to the various ethnic and geographical patterns (Garte *et al.*, 2001; Hatagima *et al.*, 2004). In our sample, the frequencies of *GSTM1* and *GSTT1* null genotypes were 0.361 and 0.331 respectively, whereas the frequency of individuals lacking both genes was 0.143 (Table 1). GST genotypes were coded as positive (wild-type homozygotes and deletion heterozygotes) or null (homozygous deletion), thereby making direct calculation of HWE impossible. Among Africans, no differences were encountered between the population studied and others analyzed for the *GSTT1* locus, whereas for *GSTM1* significant differences (p < 0.05) were found with respect to Gambia (0.200, Wild *et al.*, 2000) and Egypt (0.555, Hamdy *et al.*, 2003). In Africa, the genetic structure of many otherwise genetically “neutral” systems, such as mitochondrial DNA variation, has already been demonstrated (Salas *et al.*, 2002). Therefore, it is not surprising to discover differences between west and north African populations. The significant difference in frequency of *GTSM1*-null alleles between the Ivory Coast, Gambian and Egyptian populations could probably be attributed to diversity in populational history. Moreover, data on the prevalence of various diseases, especially those related to local exposure to toxic substances, are unavailable. Therefore, among Africans it is difficult to ascertain the cause of ethnic variation in the frequency of “null” genotypes, and the implications therefrom on the epidemiological profiles of metabolic diseases.

In conclusion, we reported on novel frequency data as regards *CYP1A1*, *GSTM1* and *GSTT1* gene polymorphisms in a north Ivory Coast population, thereby extending previous observations obtained from other African populations. Although these gene polymorphisms are not neutral, and thus cannot be used in anthropological studies as genetic markers are subject to stochastic processes, knowledge of their frequency distribution in the Ivory Coast could be helpful in understanding their roles as genetic susceptibility markers in African populations.

## Figures and Tables

**Table 1:** Genotype and allele Frequencies at *CYP1A1* and *GSTs* loci in the Ouangolodougou population (n = 133)

Genotype	Observed subjects (frequency)	Expected subjects (frequency)	χ^2^ (1 d.f.)	Allele (frequency)
*CYP1A1*				
Ile/Ile	36 (0.271)	51 (0.383)	4.41	Ile (0.617)
Ile/Val	92 (0.692)	63 (0.474)	13.35	Val (0.383)
Val/Val	5 (0.037)	19 (0.143)	10.32	
Total			28.08	
*GSTM1*				
Present	85 (0.639)			
Null	48 (0.361)			
*GSTT1*				
Present	89 (0.669)			
Null	44 (0.331)			
*GSTM1* null and *GSTT1* null	19 (0.143)			

n = number of tested individuals.

**Table 2:** Frequency of *CYP1A1* and *GSTs* null genotypes in certain African populations.

Population	n	*GSTM1* null frequency	n	*GSTT1* null frequency	Reference
Africans	114	0.330	114	0.250	Dandara *et al.* (2002)
Africans	479	0.267			Garte *et al.* (2001)
Egypt	200	0.555	200	0.295	Hamdy *et al.* (2003)
Gambia	337	0.202	326	0.371	Wild *et al.* (2000)
Ivory Coast	133	0.361	133	0.331	Present study
Tunisia	79	0.456	79	0.443	Ouerhani *et al.* (2006)

Population	n	*Ile* allele frequency			Reference

Africans	114	114	0.987		Dandara *et al.* (2002)
Africans	445	445	0.993		Garte *et al.* (2001)
African-Americans	290	290	0.969		Taioli *et al.* (1998)
African-Americans	539	539	0.970		Garte (1998)
African-Brazilians	231	231	0.855		Gaspar *et al.* (2002)
African-Brazilians	21	21	0.905		Hamada *et al.* (1995)
Benin	94	94	1.000		Jiang *et al.* (2005)
Ivory Coast	133	133	0.617		Present Study
Mali	116	116	1.000		Garte (1998)
Zimbabwe	225	225	1.000		Masimirembwa *et al.* (1998)

n = number of tested individuals.

## References

[Bajpaietal2007] Bajpai P., Tripathi A.K., Agrawal D. (2007). Increased frequencies of glutathione-S-transferase (GSTM1 and GSTT1) null genotypes in Indian patients with chronic myeloid leukaemia. Leuk Res.

[Chaetal2007] Cha I.H., Park J.Y., Chung W.Y., Choi M.A., Kim H.J., Park K.K. (2007). Polymorphisms of CYP1A1 and GSTM genes and susceptibility to oral cancer. Yonsei Med J.

[Chenetal2001] Chen S., Xue K., Xu L., Ma G., Wu J. (2001). Polymorphisms of the CYP1A1 and GSTM1 genes in relation to individual susceptibility to lung carcinoma in Chinese population. Mut Res Genom.

[Dandaraetal2002] Dandara C., Savi J., Masimirembwa C.M., Marimba A., Kaaya S., De Sommers K., Snyman J.R., Hasler J.A. (2002). Genetic polymorphism of cytochrome P450 1A1 (Cyp1A1) and glutathione trasferases (M1, T1 and P1) among Africans. Clin Chem Lab Med.

[GarriganandHammer2006] Garrigan D., Hammer M.F. (2006). Reconstructing human origins in the genomic era. Nat Rev Genet.

[Garte1998] Garte S. (1998). The role of ethnicity in cancer susceptibility gene polymorphisms: The example of CYP1A1. Carcinogenesis.

[Garteetal2001] Garte S., Gaspari L., Alexandrie A.K., Ambrosone C., Autrup H., Autrup J.L., Baranova H., Bathum L., Benhamou S., Boffetta P. (2001). Metabolic gene polymorphism frequencies in control populations. Cancer Epidem Biomar.

[Gasparetal2002] Gaspar P.A., Kvitko K., Papadpolis L.G., Hutz M.H., Weimer T.A. (2002). High frequency of CYP1A1*2C allele in Brazilian populations. Hum Biol.

[Gertigetal1998] Gertig D.M., Stampfer M., Haiman C., Hennekens C.H., Kelsey K. (1998). Glutathione S-transferase GSTM1 and GSTT1 polymorphisms and colorectal cancer risk: A prospective study. Cancer Epid Biom Prev.

[Hamadaetal1995] Hamada G.S., Sugimura H., Suzuki I., Nagura K., Kiyokawa E., Iwase T., Tanaka M., Takahashi T., Watanabe S., Kino I. (1995). The heme-binding region polymorphism of cytochrome P450IA1 (CYP1A1), rathern than the *Rsa*I polymorphism of IIE1 (CYPIIE1), is associated with lung cancer in Rio de Janeiro. Cancer Epidem Biomar.

[Hamdyetal2003] Hamdy S.I., Hiratsuka M., Narahara K., Endo N., El-Enany M., Moursi N., Ahmed M.S., Mizugaki M. (2003). Genotype and allele frequencies of TPMT, NAT2, GST, SULT1A1 and MDR-1 in the Egyptian population. Br J Clin Pharmacol.

[Hatagimaetal2004] Hatagima A., Marques C.F.S., Krieger E., Feitosa M.F. (2004). Glutathione S-transferase M1 (*GSTM1*) and T1 (*GSTT1*) polymorphisms in a Brazilian mixed population. Hum Biol.

[IndulskiandLutz2000] Indulski J.A., Lutz W. (2000). Metabolic genotype in relation to individual susceptibility to environmental carcinogenesis. Int Arch Occup Environ Health.

[Jiangetal2005] Jiang Z., Dalton T.P., Jin L., Wang B., Tsuneoka Y., Shertzer H.G., Deka R., Nebert D.W. (2005). Toward the evaluation of function in genetic variability: characterising human SNP frequencies and establishing BAC-transgenic mice carrying the human CYP1A1_CYP1A2 locus. Hum Mutat.

[Masimirembwaetal1998] Masimirembwa C.M., Dandara C., Sommers D.K., Snyman J.R., Hasler J.A. (1998). Genetic polymorphism of cytochrome P4501A1, microsomal epoxide hydrolase, and glutathione S-transferase M1 and T1 in Zimbabweans and Venda of Southern Africa. Pharmacogenetics.

[McGrathetal2007] McGrath M., Hankinson S.E., De Vivo I. (2007). Cytochrome P450 1A1, cigarette smoking, and risk of endometrial cancer. Cancer Causes Control.

[Nebertetal1996] Nebert D.W., McKinnon R.A., Puga A. (1996). Human drug-metabolizing enzyme polymorphisms: Effects on risk and toxicity of cancer. DNA Cell Biol.

[Nelsonetal1995] Nelson H.H., Wiencke J.K., Christiani D.C., Cheng T.J., Zuo Z.F., Schwartz B.S., Lee B.K., Spitz M.R., Wang M., Xu X. (1995). Ethnic differences in the prevalence of the homozygous deleted genotype of glutathione S-transferase theta. Carcinogenesis.

[Ouerhanietal2006] Ouerhani S., Tebourski F., Slama M.R., Marrakchi R., Rabeh M., Hassine L.B., Ayed M., Elgaaïed A.B. (2006). The role of glutathione transferases M1 and T1 in individual susceptibility to bladder cancer in a Tunisian population. Ann Hum Biol.

[Pembleetal1994] Pemble S., Schroeder K.R., Spenser S.R. (1994). Human glutathione-S-transferase theta (GSTT1): cDNA cloning and the characterization of a genetic polymorphism. Biochem J.

[Perera1997] Perera F.P. (1997). Environment and cancer: Who are susceptible?. Science.

[RaymondandRousset1995] Raymond M., Rousset F. (1995). GENEPOP v. 1.2: Population genetics software for exact tests and ecumenicism. J Hered.

[Royetal2001] Roy B., Majumder P.P., Dey B., Chakraborty M., Banerjee S., Roy M., Mukherjee N., Sil S.K. (2001). Ethnic differences in distributions of GSTM1 and GSTT1 homozygous “null” genotypes in India. Hum Biol.

[Salasetal2002] Salas A., Richards M., De la Fe T., Lareu M.V., Sobrino B., Sánchez-Diz P., Macaulay V., Carracedo A. (2002). The making of the African mtDNA landscape. Am J Hum Genet.

[Taiolietal1998] Taioli E., Ford J., Trachman J., Li Y., Demopoulos R., Garte S. (1998). Lung cancer risk and CYP1A1 genotype in African Americans. Carcinogenesis.

[Walshetal1991] Walsh P.S., Metzger D.A., Higuchi R. (1991). Chelex 100 as a Medium for simple extraction of DNA for PCR-based typing from forensic material. BioTechniques.

[Wildetal2000] Wild C.P., Yin F., Turner P.C., Chemin I., Chapot B., Mendy M., Whittle H., Kirk G.D., Hall A.J. (2000). Environmental and genetic determinants of aflatoxin-albumin adducts in the Gambia. Int J Cancer.

[Zhongetal1993] Zhong S., Wyllie A.H., Barnes D., Wolf C.R., Spurr N.K. (1993). Relationship between the GSTM1 genetic polymorphism and susceptibility to bladder, breast and colon cancer. Carcinogenesis.

